# PReS-FINAL-2362: The spectrum of childhood inflammatory brain diseases; an increasingly recognised field

**DOI:** 10.1186/1546-0096-11-S2-P352

**Published:** 2013-12-05

**Authors:** M Twilt, S Sheikh, S Benseler, All the members of the Brainworks Investigator group

**Affiliations:** 1 rheumatology, the hospital for sick children, toronto, Canada; 2 rheumatology, Birmingham children's hospital, Birmingham, UK

## Introduction

Childhood Inflammatory Brain diseases are potentially life-threatening diseases leading to severe neurological deficits if not treated. The spectrum of childhood inflammatory brain diseases is rapidly expanding.

## Objectives

To review all pediatric patients enrolled in the international BrainWorks database.

## Methods

All pediatric patients enrolled through a BrainWorks center were identified in the BrainWorks database. Patients were eligible for inclusion if predetermined information at the baseline visit was available. This included, information on demographic information, such as age at diagnosis and gender, diagnosis at enrollment, clinical, laboratory, neuroradiology and if applicable brain biopsy.

## Results

In total 247 (132 boys, 115 girls) Canadian children were enrolled in BrainWorks with complete dataset at baseline. The mean age at diagnosis was 9.33 years (boys 9.67 years, girls 9.07 years). The top 4 diagnosis included were; Non-progressive large vessel CNS vasculitis n = 90 (63 boys, 27 girls, mean age 8.15 years), Small vessel CNS vasculitis n = 57 (19 boys, 38 girls, mean age 11 years), Progressive large vessel CNS vasculitis n = 25 (21 boys, 4 girls, mean age 10.3 years) and NMDAR-encephalitis n = 25 (7 males, 18 females, mean age 9.9 years). Other diagnosis included CNS vasculitis due to infection n = 13, CNS vasculitis in children with an underlying rheumatic disease n = 10 or systemic vasculitis n = 6, other neuronal antibody mediated diseases n = 6, ADEM n = 5, channelopathies n = 2, and other vasculopathies n = 8. Focal deficits at presentation were more commonly seen in patients with large vessel CNS vasculitis (79% non-progressive, 84% progressive), while diffuse deficits were seen more in children with small vessel CNS vasculitis and NMDAR encephalitis (61& and 72%). Seizures were seen in all inflammatory brain diseases, but were more frequently present in NMDAR encephalitis and small vessel CNS vasculitis (80% and 61%) compared to the large vessel CNS vasculitis subytpes (non-progressive 18%, progressive 18%).

## Conclusion

Childhood inflammatory Brain diseases encompasses many different diagnosis. The most frequent diagnosis are the different subtypes of childhood CNS vasculitis, however NMDAR encephalitis is increasingly recognized and diagnosed.

## Disclosure of interest

None declared.

